# Exploring the causal relationship between gut microbiota and thromboembolism: A Mendelian randomization study

**DOI:** 10.1097/MD.0000000000045790

**Published:** 2025-11-14

**Authors:** Zhiyi Wang, Zhijun Zheng

**Affiliations:** a Dongguan SongShan Lake Central Hospital, Dongguan, Guangdong Province, China; b Graduate School of Jiangxi University of Traditional Chinese Medicine, Nanchang, Jiangxi Province, China.

**Keywords:** arterial embolism and thrombosis, causal relationship, gut microbiota, Mendelian randomization analysis, pulmonary embolism, thromboembolism, venous thromboembolism

## Abstract

Although several studies have indicated potential associations between gut microbiota (GM) and thromboembolic diseases, the causative role of GM in thromboembolism remains unclear. We employed two-sample Mendelian randomization (MR) analysis to investigate the potential causal association between GM and 3 thromboembolic conditions: arterial embolism and thrombosis (AET), venous thromboembolism (VTE), and pulmonary embolism (PE). Genome-wide association study datasets from MiBioGen and FinnGen were utilized, with GM serving as the exposure and thromboembolism outcomes as endpoints. Using the inverse-variance weighted approach, under conditions without heterogeneity or horizontal pleiotropy, 16 gut microbial genera were significantly associated with thromboembolic risks. Specifically, *Holdemanella* and the *Ruminococcus gnavus group* exhibited protective effects against AET, whereas *Parabacteroides*, *Subdoligranulum*, and the *Eubacterium hallii group* were linked to elevated AET risk. Regarding VTE, protective associations were found for *Ruminococcaceae NK4A214 group*, *Ruminococcaceae UCG002*, *Ruminococcaceae UCG004*, *Lachnospiraceae UCG010*, *Sutterella*, and *Christensenellaceae R.7 group*, while the *Eubacterium rectale group* showed a positive correlation with increased VTE risk. Furthermore, *Defluviitaleaceae UCG011*, *Ruminococcaceae UCG004*, *Turicibacter*, *Lachnospiraceae UCG010*, *Sutterella*, and *Collinsella* demonstrated protective effects against PE, whereas *Erysipelatoclostridium* was positively associated with heightened PE risk. In conclusion, this two-sample MR study suggests potential associations between GM and thromboembolic disorders. However, as none of the associations remained significant after Bonferroni correction, these findings should be regarded as exploratory and hypothesis-generating, requiring further validation in future studies.

## 1. Introduction

Thromboembolism refers to a disease where a solid mass in the blood obstructs a blood vessel.^[1]^ Thrombosis can develop in either arteries or veins,^[1]^ and depending on the site and vessel type, thromboembolism can result in diverse clinical presentations and health complications. Arterial embolism and thrombosis (AET), which occurs within arteries, is commonly triggered by vascular endothelial injury, platelet activation, and the coagulation system’s activation due to atherosclerosis.^[2,3]^ It primarily affects the coronary arteries and cerebral arteries, leading to myocardial infarction and ischemic stroke.^[4]^ Venous thromboembolism (VTE) is characterized by the development of blood clots in the venous system, frequently presenting as deep vein thrombosis, primarily located in the DV of the legs. Virchow triad identifies venous stasis, endothelial injury, and hypercoagulability as the fundamental mechanisms driving the pathogenesis of VTE.^[5,6]^ Importantly, deep vein thrombosis can progress to pulmonary embolism (PE), a severe complication characterized by acute chest pain, respiratory distress, and potential fatality.^[7–9]^ Current clinical management strategies for thromboembolic disorders largely consist of anticoagulant pharmacotherapy and surgical interventions.^[10]^ However, prolonged anticoagulant use may increase hemorrhagic risk and adversely affect organ function,^[11]^ while surgical approaches involve inherent procedural risks. Consequently, ongoing medical research continues to prioritize improvements in both thromboembolism treatment and prevention.

The gut microbiota (GM) exerts a fundamental influence on host physiological homeostasis,^[12,13]^ and accumulating evidence has associated GM alterations with various systemic disorders including AET,^[14,15]^ VTE, and PE. GM dysbiosis may provoke systemic inflammatory responses, a known critical contributor to thrombosis.^[16,17]^ Additionally, alterations in GM composition might elevate thrombosis risk via immune modulation and functional impairment of vascular endothelium.^[18–22]^ Notably, observational clinical studies examining associations between GM and thromboembolic conditions are prone to confounding effects arising from environmental factors and dietary variations.^[23]^

Mendelian randomization (MR) denotes a genetic instrumental variable analysis that employs genetic variants, particularly single nucleotide polymorphisms (SNPs), as instruments linked to exposure factors. The fundamental concept of MR involves deducing causal connections between exposures and outcomes by utilizing the genetic association of instrumental SNPs with the outcomes being studied. SNPs are prevalent genetic variants distributed randomly across populations and inherited stably, thereby minimizing potential biases from confounding variables and bolstering the internal validity of causal inference studies.^[24]^ Therefore, this investigation employed genome-wide association study (GWAS) datasets obtained from MiBioGen and FinnGen R10, selected SNPs associated with GM composition as genetic instruments, and applied MR analyses to elucidate causal associations between GM and thromboembolic disorders. This approach provides hypothesis-generating evidence and offers novel insights that may inform future mechanistic studies and potential therapeutic strategies.

## 2. Materials and methods

### 2.1. Study overview

The results of this study were reported in accordance with the recommendations made by the STROBE-MR.^[25,26]^

**Figure 1.**
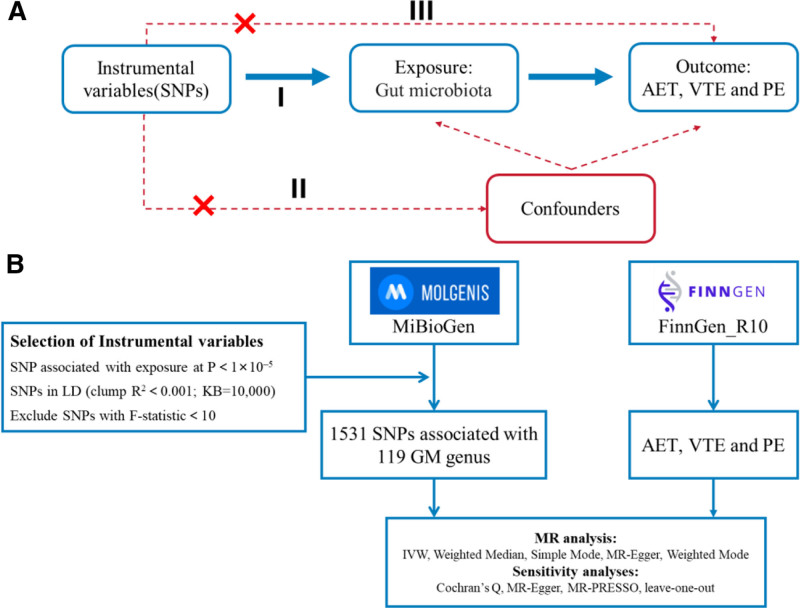
(A) Three fundamental assumptions underpinning MR: I – correlation assumption; II – independence assumption; III – exclusion restriction assumption; (B) Detailed workflow of the MR analysis used in this study. MR = Mendelian randomization.

Figure 1 provides a schematic representation of the research workflow. Extensive GWAS data concerning GM and thromboembolic conditions were collected, with each microbial genus considered as a unique exposure variable, while the 3 types of thromboembolism were analyzed individually as outcome variables. A two-sample MR analysis was performed to assess possible causal relationships between GM and the 3 thromboembolic outcomes. To ensure reliable conclusions, 3 fundamental assumptions of MR needed to be fulfilled: Instrumental variable SNPs demonstrated a strong correlation with the exposure (GM); the chosen SNPs showed no relationship with confounding variables; and these SNPs influenced thromboembolism risk exclusively through their connection with GM, with no other causal pathways identified.^[27,28]^ (Fig. 1).

### 2.2. Data sources

The MR analysis used data from 2 separate GWAS datasets. The MiBioGen collaboration, which included 18,340 people from a variety of ethnic backgrounds, was the source of the GM data.^[29]^ Initially, 14,587 GM-related SNPs identified from human samples met the significance criterion (*P* < 1 × 10^−5^); following exclusion of SNPs corresponding to 12 unidentified genera, 119 genera remained eligible for inclusion.^[29]^ Data concerning AET, VTE, and PE were obtained from the FinnGen R10 dataset.^[30]^ Specifically, the AET cohort comprised 383,860 participants (1883 cases and 381,977 controls), encompassing 21,305,815 SNPs. The VTE cohort included 412,181 individuals (21,021 cases, 391,160 controls) with 21,306,349 SNPs, while the PE dataset encompassed 411,174 participants (10,046 cases, 401,128 controls), containing 23,106,331 SNPs. All participants were of European descent. Public GWAS summary data utilized herein had received prior ethical approval from their respective institutions. Table 1 provides further detailed descriptions (Table 1).

**Table 1:** Details of the genome-wide association studies included in the Mendelian randomization analysis.

Trait	Data type	N_cases	N_controls	Consortium
GM	Exposure	18,340		MiBioGen
AET	Outcome	1883	381,977	FinnGen_R10
VTE	Outcome	21,021	391,160	FinnGen_R10
PE	Outcome	10,046	401,128	FinnGen_R10

AET = arterial embolism and thrombosis, GM = gut microbiota, PE = pulmonary embolism, VTE = venous thromboembolism.

### 2.3. Selection of instrumental variables (IVs)

The inferred causal links between GM and thromboembolism were made more reliable with the implementation of strict quality control measures. At first, SNPs linked to GM were chosen as IVs using a strict significance threshold (*P* < 1 × 10^−5^).^[31]^ Subsequently, linkage disequilibrium pruning using European genomic reference panels (kb = 10,000, *r*^2^ < 0.001) was conducted to ensure SNP independence.^[32]^ Palindromic SNPs were removed to minimize potential allele-orientation bias. Moreover, SNPs associated with both exposure and outcome factors were matched, and effect alleles were aligned for data accuracy. Lastly, the strength and reliability of selected IVs were evaluated through *F*-statistics, calculated as *F* = β^2^_exposure/ SE^2^_exposure.^[29]^ IVs demonstrating *F*-statistics ≤10 were excluded to mitigate weak instrument bias.

### 2.4. Statistical methods and sensitivity analysis

This study utilized 5 complementary MR analytical methods – inverse-variance weighted (IVW), Weighted median, Simple mode, MR-Egger, and Weighted mode – to systematically examine the potential causal relationships between GM and thromboembolic diseases.^[33]^ Among these, the IVW analysis functioned as the primary analytical approach.^[34]^ To account for multiple testing and reduce the likelihood of false-positive results, a rigorous Bonferroni correction was implemented, establishing a significance threshold of *P* = 4.20 × 10^−4^ (0.05/119).^[35]^

The evaluation of heterogeneity among MR estimates was conducted using Cochran *Q* test. A non-significant result (*P* > .05) suggested that significant heterogeneity was not present. Based on the outcomes of the heterogeneity assessment, a decision was made to utilize either a fixed-effects or random-effects model. The assessment of potential horizontal pleiotropy (HP) was conducted through MR-Egger intercept and MR-PRESSO analyses. A non-significant intercept (*P* > .05) suggested the absence of pleiotropy, thus confirming the robustness of the findings. Furthermore, leave-one-out (LOO) sensitivity analyses were performed to systematically exclude each SNP and assess its influence on the overall MR estimate, thereby improving the robustness of the results. Funnel plots and forest plots offered visual evaluations of the consistency and stability of the MR estimates. All statistical procedures were executed using *R*-4.3.2 and RStudio software, employing the TwoSampleMR package (version 0.5.7).

## 3. Results

### 3.1. IV selection

**Figure 2.**
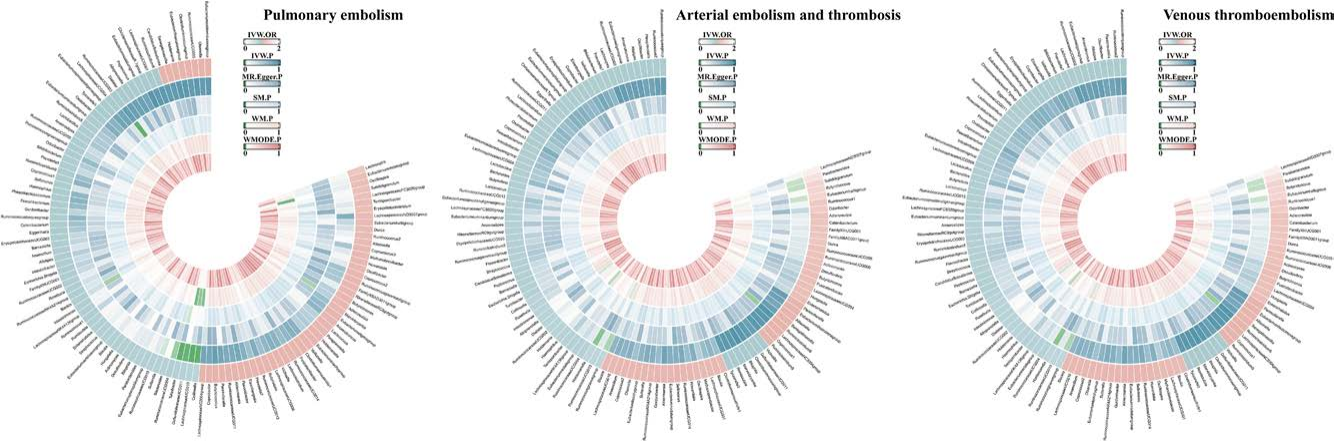
Circular plot summarizing MR analysis outcomes for all GM genera. GM = gut microbiota, MR = Mendelian randomization.

After applying stringent selection criteria for IVs as outlined in Section 2.3, we identified 1531 SNPs linked to 119 genera of GM, all demonstrating strong instrument strength with *F*-statistics exceeding 10,^[36]^ thereby reducing the potential for weak instrument bias (Table S1, Supplemental Digital Content, https://links.lww.com/MD/Q624). Figure 2 illustrates the causal relationships deduced from MR analyses involving the 119 GM genera and 3 thromboembolic disorders (Tables S2–S4, Supplemental Digital Content, https://links.lww.com/MD/Q624)(Fig. 2).

### 3.2. The causal relationship between GM and 3 types of thromboembolism

#### 3.2.1. The causal relationship between GM and AET

**Figure 3.**
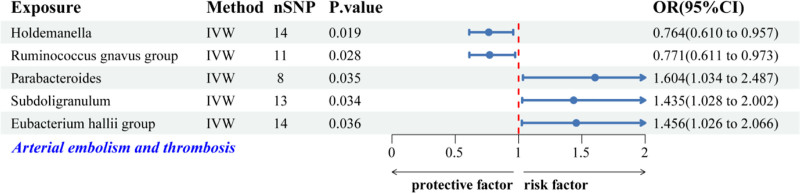
Forest plot illustrating associations between genetically predicted GM genera and AET risk based on IVW analysis. AET = arterial embolism and thrombosis, GM = gut microbiota, IVW = inverse-variance weighted.

**Figure 4.**
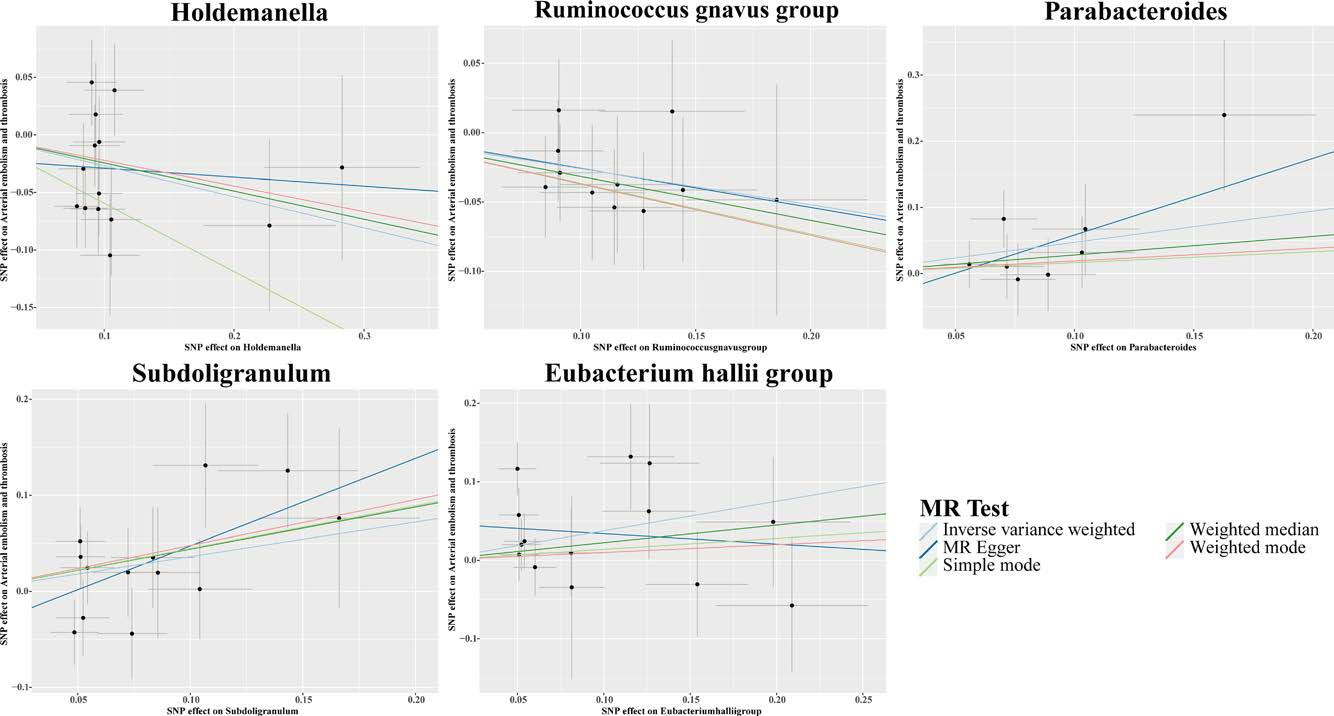
Scatter plots for causal effects of GM on AET risk using 5 MR methods. AET = arterial embolism and thrombosis, GM = gut microbiota, MR = Mendelian randomization.

The IVW MR analyses revealed connections between 5 GM genera and the likelihood of AET. In this analysis, *Holdemanella* and *Ruminococcus gnavus* group exhibited protective associations with AET risk. Conversely, *Parabacteroides*, *Subdoligranulum*, and *Eubacterium hallii group* were positively correlated with an increased susceptibility to AET (Fig. 3). Figure 4 illustrates scatter plots that represent the effect estimates of individual SNPs on the risk associated with these GM genera in relation to AET.

Furthermore, the findings from Cochran *Q*, MR-Egger, and MR-PRESSO tests revealed no indication of substantial heterogeneity or HP among the 5 genera linked to AET (Table 2). Additionally, the LOO sensitivity analyses, funnel plots, and forest plots consistently indicated the absence of significant outliers, thereby reinforcing the robustness of the MR findings (Fig. S1A–C, Supplemental Digital Content, https://links.lww.com/MD/Q623). Following the application of Bonferroni correction, it was observed that none of the identified associations attained statistical significance, indicating a need for careful interpretation of the causal relationships between GM and AET (Figs. 3 and 4, Table 2).

**Table 2:** Sensitivity analysis of the MR analysis results of the GM and AET.

Exposure	Heterogeneity		Directional pleiotropy		MR-PRESSO
	Cochran *Q*	*P*-value	Egger intercept	*P*-value	*P*-value
Holdemanella	15.084	.237	−0.021	.598	.321
Ruminococcus gnavus group	3.241	.954	0.002	.970	.977
Parabacteroides	4.474	.613	−0.057	.434	.671
Subdoligranulum	10.424	.493	−0.044	.220	.449
Eubacterium hallii group	14.880	.248	0.047	.123	.158

AET = arterial embolism and thrombosis, GM = gut microbiota, MR = Mendelian randomization.

#### 3.2.2. The causal relationship between GM and VTE

**Figure 5.**
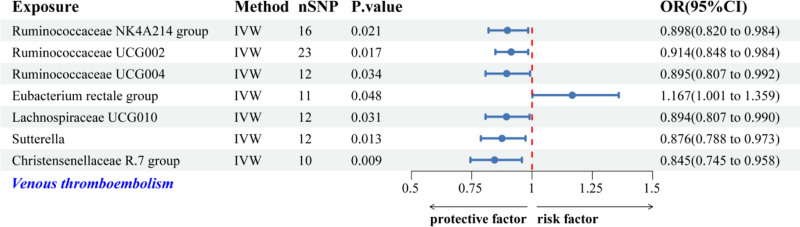
Forest plot illustrating the IVW-based associations between genetically predicted GM genera and VTE risk. GM = gut microbiota, IVW = inverse-variance weighted, VTE = venous thromboembolism.

**Figure 6.**
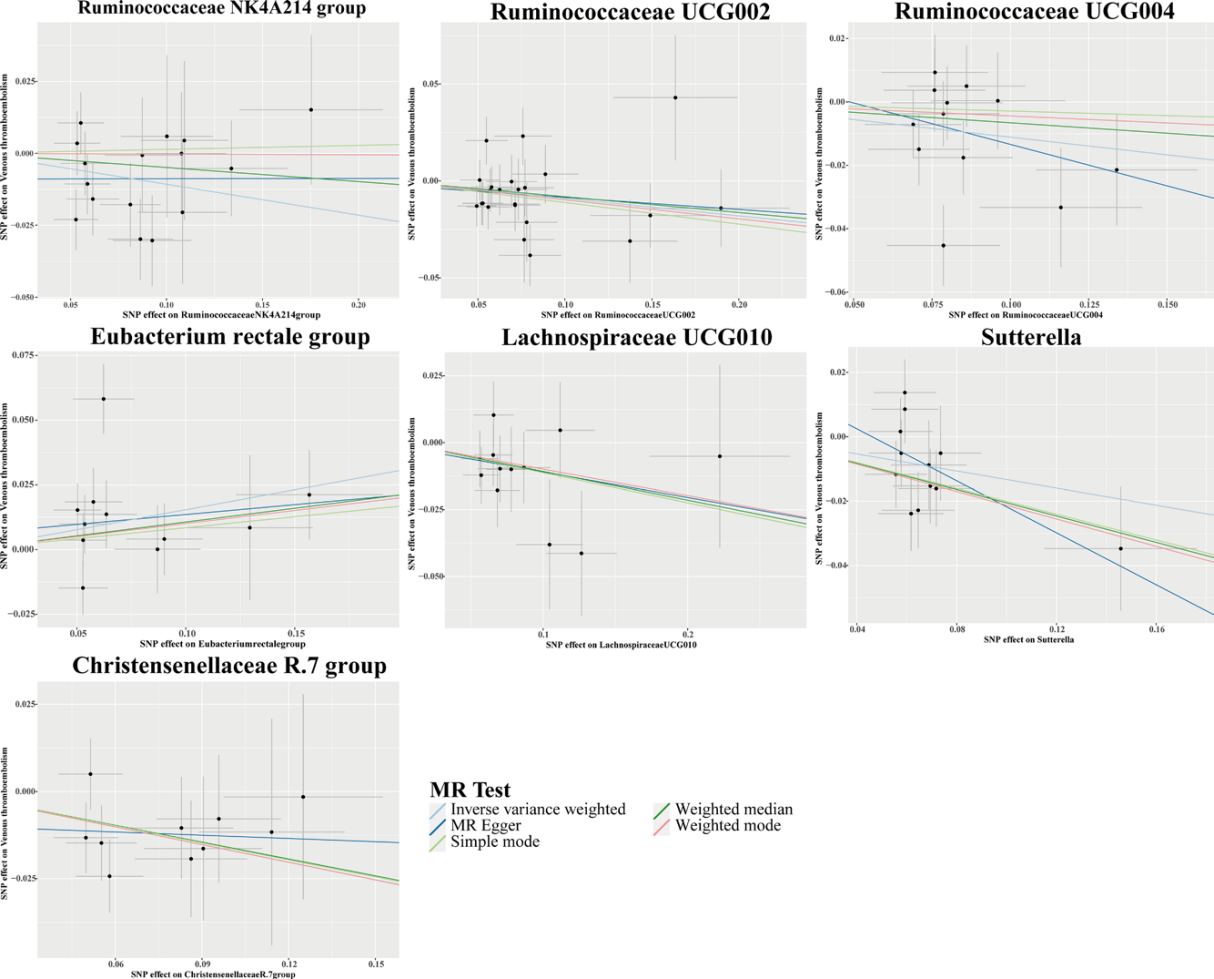
Scatter plots for causal effects of GM on VTE risk using 5 MR methods. GM = gut microbiota, MR = Mendelian randomization, VTE = venous thromboembolism.

The IVW analysis revealed 7 GM genera linked to the risk of VTE. Protective associations were identified for the *Ruminococcaceae NK4A214 group*, *Ruminococcaceae UCG002*, *Ruminococcaceae UCG004*, *Lachnospiraceae UCG010*, *Sutterella*, and *Christensenellaceae R.7 group*. Conversely, a heightened risk of VTE was linked to the *Eubacterium rectale group* (Fig. 5). Figure 6 displays scatter plots that illustrate the effect estimates of the GM-associated SNPs on the risk of VTE.

The findings from Cochran *Q* test, MR-Egger, and MR-PRESSO analyses revealed heterogeneity and HP concerning the association with the *Eubacterium rectale group (P* < .05), which may affect the validity of the results. Following the removal of 2 outlier SNPs (rs10248854 and rs3980709), the ensuing sensitivity analyses demonstrated a reduction in heterogeneity and HP (*P* < .05). The 6 genera that remain showed no signs of HP (Table 3). Additionally, LOO analyses, funnel plots, and forest plots indicated the absence of significant outliers, reinforcing the reliability of these MR findings (Fig. S2A–C, Supplemental Digital Content, https://links.lww.com/MD/Q623). Nonetheless, following the implementation of the Bonferroni correction, all associations between GM and VTE lost their statistical significance (Figs. 5 and 6, Table 3).

**Table 3:** Sensitivity analysis of the MR analysis results of the GM and VTE.

Exposure	Heterogeneity		Directional pleiotropy		MR-PRESSO
	Cochran *Q*	*P*-value	Egger intercept	*P*-value	*P*-value
Ruminococcaceae NK4A214 group	13.411	.494	−0.009	.412	.521
Ruminococcaceae UCG002	21.381	.436	−0.002	.773	.542
Ruminococcaceae UCG004	15.826	.105	0.013	.636	.153
Eubacterium rectale group	20.183	.017	0.006	.717	.041
	2.048	.957	0.009	.480	.966
Lachnospiraceae UCG010	6.713	.752	−0.001	.917	.814
Sutterella	10.838	.370	0.019	.248	.344
Christensenellaceae R.7 group	4.646	.795	−0.010	.546	.820

GM = gut microbiota, MR = Mendelian randomization, VTE = venous thromboembolism.

#### 3.2.3. The causal relationship between GM and PE

**Figure 7.**
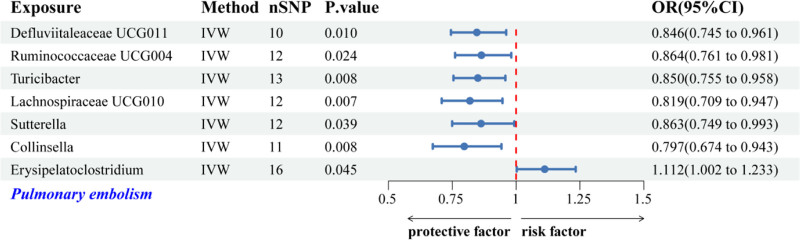
Forest plot displaying IVW-based associations between genetically predicted GM genera and the risk of PE. GM = gut microbiota, IVW = inverse-variance weighted, PE = pulmonary embolism.

**Figure 8.**
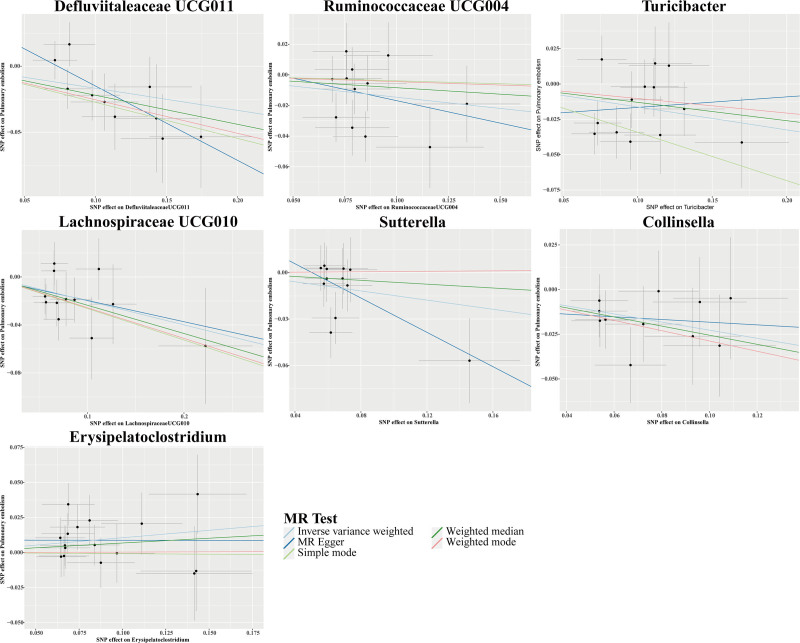
Scatter plots for causal effects of GM on PE risk using 5 MR methods. GM = gut microbiota, MR = Mendelian randomization, PE = pulmonary embolism.

The IVW analysis identified 7 GM genera associated with PE risk. Among these genera, *Defluviitaleaceae UCG011*, *Ruminococcaceae UCG004*, *Turicibacter*, *Lachnospiraceae UCG010*, *Sutterella*, and *Collinsella* exhibited protective associations against PE. In contrast, *Erysipelatoclostridium* was positively correlated with increased PE risk (Fig. 7). Scatter plots illustrating the estimated effects of GM-related SNPs on PE risk are shown in Figure 8.

The Cochran *Q* test was employed to examine the heterogeneity present among SNPs, while MR-Egger intercept and MR-PRESSO analyses were performed to investigate HP.^[33]^ The analyses conducted showed no signs of heterogeneity or HP concerning the 7 GM genera linked to PE (all *P*-values > .05; Table 4). Additionally, sensitivity assessments such as LOO analyses, funnel plots, and forest plots revealed no significant outliers,^[33]^ thereby affirming the robustness and dependability of the MR results (Fig. S3A–C, Supplemental Digital Content, https://links.lww.com/MD/Q623). However, following the application of Bonferroni correction for multiple comparisons, none of the identified causal relationships between GM and PE achieved statistical significance (Figs. 7 and 8, Table 4).

**Table 4:** Sensitivity analysis of the MR analysis results of the GM and PE.

Exposure	Heterogeneity		Directional pleiotropy		MR-PRESSO
	Cochran *Q*	*P*-value	Egger intercept	*P*-value	*P*-value
Defluviitaleaceae UCG011	4.162	.842	0.041	.134	.701
Ruminococcaceae UCG004	12.055	.281	0.012	.708	.365
Turicibacter	12.986	.294	−0.024	.367	.367
Lachnospiraceae UCG010	7.356	.691	−0.002	.921	.793
Sutterella	7.663	.662	0.028	.208	.611
Collinsella	3.152	.958	−0.011	.656	.986
Erysipelatoclostridium	9.193	.819	0.009	.612	.847

GM = gut microbiota, MR = Mendelian randomization, PE = pulmonary embolism.

## 4. Discussion

This research marks the initial exploration employing MR analysis to investigate possible causal relationships between GM and 3 categories of thromboembolic conditions: AET, VTE, and PE. Through thorough MR and sensitivity analyses encompassing 119 bacterial genera, we identified 16 genera that showed suggestive associations with thromboembolism risk. Sensitivity analyses indicated no major heterogeneity or pleiotropy, supporting the robustness of these exploratory findings. These results provide hypothesis-generating evidence regarding the potential link between GM composition and thromboembolic disorders.

Specifically, our findings indicated that increased abundances of *Parabacteroides*, *Subdoligranulum*, and the *Eubacterium hallii group* appeared to be associated with a higher risk of AET. Previous evidence suggests *Parabacteroides* plays an essential role in inflammation, a fundamental trigger for embolic events.^[37]^ Furthermore, observational data indicate that *Subdoligranulum* is implicated as a risk factor for vasculitis,^[38]^ a condition linked to endothelial damage and subsequent arterial thrombosis. Similarly, increased presence of the *Eubacterium hallii group* has been observed among patients with cirrhosis complicated by portal vein thrombosis,^[39]^ aligning with the elevated AET risk reported here. Although the context of the previous study was cirrhosis with portal vein thrombosis, this result indirectly suggests that the impact of the *Eubacterium hallii group* on thrombosis formation might not be limited to specific types of vessels or disease contexts. Therefore, the mechanism by which the *Eubacterium hallii group* promotes thrombosis formation warrants further investigation. Additionally, *Holdemanella* and the *Ruminococcus gnavus* group are protective factors against AET. They may enhance the host’s immune function, likely due to the production of butyrate and isovalerate.^[40,41]^

Our study found that the risk of VTE may be positively correlated with the *Eubacterium rectale group*. Currently, there are no reports in the literature linking the *Eubacterium rectale group* with VTE; however, the complex interactions between GM and the host may influence thrombus formation. Intriguingly, earlier research has shown that the *Eubacterium rectale group* may modulate host inflammation through metabolic interactions.^[42]^ Enhanced abundance of this group can disrupt the Th17/Treg balance, driving systemic inflammation and potentially heightening VTE risk,^[42]^ a mechanistic pathway warranting further investigation. In contrast, we observed protective associations against VTE for various genera, such as the *Ruminococcaceae NK4A214 group*, *Ruminococcaceae UCG002*, *Ruminococcaceae UCG004*, *Lachnospiraceae UCG010*, *Sutterella*, and *Christensenellaceae R.7 group*. These genera probably influence their protective mechanisms by generating short-chain fatty acids, including butyrate and acetate, which are recognized for their anti-inflammatory characteristics and their involvement in the metabolism of complex polysaccharides.^[43–45]^

Our analysis further indicated *Erysipelatoclostridium* showed a suggestive association with increased PE risk. This genus interacts with host Toll-like receptors, subsequently initiating signaling cascades that promote inflammatory cytokine release, including IL-6 and IL-1β, potentially exacerbating thromboembolic conditions.^[44,46–48]^ Another clinical study found that *Erysipelatoclostridium* is positively correlated with vascular diseases, which can increase the risk of thrombosis.^[49]^ Conversely, *Defluviitaleaceae UCG011*, *Ruminococcaceae UCG004*, *Turicibacter*, *Lachnospiraceae UCG010*, *Sutterella*, and *Collinsella* were found to reduce the risk of PE, suggesting that they may be protective factors. Microorganisms from the *Ruminococcaceae* and *Lachnospiraceae* families possess the capability to ferment dietary fibers, resulting in the production of short-chain fatty acids. These metabolites are acknowledged for their roles in diminishing systemic inflammation, strengthening the integrity of the gut barrier, and influencing immune responses.^[43]^ Moreover, genera such as *Turicibacter* and *Sutterella* are implicated in immune regulation pathways, potentially mitigating excessive inflammation and maintaining immune homeostasis, thereby lowering the risk of PE.^[43,50]^

It is well established that PE is typically caused by a VTE that breaks free and travels through the bloodstream to the pulmonary vessels.^[7]^ In our study, an interesting phenomenon was observed: *Ruminococcaceae UCG004*, *Lachnospiraceae UCG010*, and *Sutterella* were protective factors in both PE and VTE. This finding indicates that these bacterial groups may represent exploratory microbial markers relevant to thrombotic disease research, though further validation is needed before any preventive implications can be drawn.

Employing MR methodology, we elucidated potential causal links between GM composition and 3 thromboembolic conditions. This research offers several strengths. Firstly, the MR approach utilized genetic variants as IVs, effectively controlling for potential confounders such as lifestyle and environmental influences. Secondly, the latest data from the Finnish R10 database significantly enhanced the statistical robustness and precision of our conclusions. Lastly, comprehensive sensitivity analyses assured the reliability and consistency of our findings.

Nevertheless, several limitations should be acknowledged. First, the analyses exclusively involved individuals of European ancestry, which may limit the generalizability of the findings to other populations. Second, our investigation was confined to genus-level GM, lacking higher taxonomic resolution. Third, available GWAS data did not allow stratification according to demographic factors such as age or gender. Fourth, no formal power calculation was conducted, which may limit the ability to detect modest causal effects. Future MR studies with larger datasets should incorporate power assessments to ensure adequate sensitivity. Finally, after Bonferroni correction for multiple testing, none of the observed associations remained statistically significant; therefore, the results should be regarded as exploratory and hypothesis-generating. Furthermore, future studies should incorporate formal power calculations to ensure adequate sensitivity, expand the scope of investigation to include diverse populations beyond European ancestry, and conduct higher-resoluti.

## 5. Conclusion

In conclusion, this two-sample MR study explored potential associations between GM and 3 thromboembolic disorders, with several bacterial genera showing suggestive protective or risk effects. These findings should be regarded as exploratory and hypothesis-generating, requiring validation in future large-scale studies.

## Acknowledgments

MiBioGen and FinnGen database were used in this study, and the authors would like to thank all those who contributed and participated in the data collection.

## Author contributions

**Data curation:** Zhiyi Wang, Zhijun Zheng.

**Investigation:** Zhiyi Wang.

**Methodology:** Zhiyi Wang.

**Resources:** Zhijun Zheng.

**Software:** Zhiyi Wang, Zhijun Zheng.

**Validation:** Zhijun Zheng.

**Writing – original draft:** Zhiyi Wang.

**Writing – review & editing:** Zhijun Zheng.

## Supplementary Material




