# Supplementary material for: Exploring the causal relationship between gut microbiota and thromboembolism: A Mendelian randomization study
Source: Medicine (Baltimore). 2025 Nov 14;104(46):e45790. doi: 10.1097/MD.0000000000045790 (PMC12622754; doi:10.1097/MD.0000000000045790)
Supplement: Supplementary file 2 [file medi-104-e45790-s002.docx]

**Supplementary Figure S1.** Causal estimates of exposure (Specific GM) on AET. A): Leave-one-out stability tests, Calculate the MR results of the remaining IVs after removing the IVs one by one; C): Funnel plots; D): Forest plots





**Supplementary Figure S2.** Causal estimates of exposure (Specific GM) on VTE. A): Leave-one-out stability tests, Calculate the MR results of the remaining IVs after removing the IVs one by one; C): Funnel plots; D): Forest plots.





**Supplementary Figure S3.** Causal estimates of exposure (Specific GM) on PE. A): Leave-one-out stability tests, Calculate the MR results of the remaining IVs after removing the IVs one by one; B): Funnel plots; C): Forest plots.
